# Multi-omics analysis reveals the effects of microbiota on oral homeostasis

**DOI:** 10.3389/fimmu.2022.1005992

**Published:** 2022-09-20

**Authors:** Huiqing Long, Li Yan, Juncai Pu, Yiyun Liu, Xiaogang Zhong, Haiyang Wang, Lu Yang, Fangzhi Lou, Shihong Luo, Yingying Zhang, Yang Liu, Peng Xie, Ping Ji, Xin Jin

**Affiliations:** ^1^ Key Laboratory of Psychoseomadsy, Stomatological Hospital of Chongqing Medical University, Chongqing, China; ^2^ Chongqing Key Laboratory of Oral Diseases and Biomedical Sciences, Chongqing, China; ^3^ School of Public Health and Management, Chongqing Medical University, Chongqing, China; ^4^ NHC Key Laboratory of Diagnosis and Treatment on Brain Functional Diseases, The First Affiliated Hospital of Chongqing Medical University, Chongqing, China

**Keywords:** microbiota, tongue, homeostasis, immunity, transcriptome, proteome, metabolome

## Abstract

The oral epithelium’s normal morphological structure and function play an important role in maintaining oral homeostasis, among which microbiota and chronic stress are key contributing factors. However, the effects of microbiota and chronic stress on the morphological structures and molecular function of oral homeostasis remain unclear. In this study, morphological staining was used to compare the tongue structure of specific pathogen-free and germ-free mice, and an integrated multi-omics analysis based on transcriptomics, proteomics, and metabolomics was performed to investigate the regulatory mechanisms of microbiota and chronic stress on oral homeostasis. We found that the morphological structure of the tongue in germ-free mice was disordered compared with in specific pathogen-free mice, especially in the epithelium. Multi-omics analysis indicated that differentially expressed molecules of the tongue between germ-free and specific pathogen-free mice were significantly enriched in the mitochondrial metabolic process and immune response. Interestingly, microbiota also significantly influenced the permeability of the oral epithelial barrier, represented by the differential expression of keratinization, and cell adhesion molecules. It was worth noting that the above changes in the tongue between specific pathogen-free and germ-free mice were more significant after chronic stress. Collectively, this is the first study to reveal that the microbiota might maintain oral homeostasis by reshaping the structure of the oral epithelial barrier and changing the function of molecular biology, a process that may be driven by the immune response and mitochondrial metabolic process of oral tissue. Furthermore, chronic stress can enhance the regulatory effects of microbiota on oral homeostasis.

## Introduction

The mouth is an organ that communicates directly with external pathogens and is exposed to the unstable microenvironment formed by various bacteria, viruses and fungi for a long time (1). A delicate balance between the host and oral microbiota is established, known as oral homeostasis, and heredity and the external environment may disrupt this natural balance in the mouth, resulting in regional oral and systemic diseases. The microbiota and chronic stress are key influencing factors in the acquired environment.

The interaction between microbiota and host plays an important role in maintaining homeostasis. Compared with conventionally fed mice, the intestinal surface area and villi number of germ-free (GF) mice are reduced, accompanied by decreased immune function (2). Similar findings were found in the nasal mucosa of GF mice (3). Research on microbiota in stomatology has gained significant momentum in recent years. Most oral diseases such as periodontitis and oral squamous cell carcinoma are closely related to the dysbiosis of oral microbiota (4, 5). However, the above studies have been mainly focused on the interaction between microbiota and certain oral diseases, with no emphasis placed on the effect and mechanism of microbiota on the morphological structure and biological function of oral tissue from an integrated multi-omics perspective.

Stress related factors that cause psychological anxiety and depression are closely related to oral diseases (6). Many oral diseases are accompanied by a high incidence of anxiety and depression (7, 8), and the prevalence of depression in oral squamous cell carcinoma reportedly exceeds 65% (9). Meanwhile, current evidence suggests that bad mental attitudes are not conducive to the prognosis of oral disease (10), while inflammatory mediators were found to be increased locally in the tumors of stressed rats (11). Moreover, it has been found that the expression of neurotransmitters was elevated in saliva and serum of patients with burning mouth syndrome with mood disorders (12). Although significant emphasis has been placed on the interaction between stress-related factors and oral diseases, few studies have investigated the potential molecular mechanisms.

This study sought to provide an integrated multi-omics perspective to explore the effects of microbiota on oral homeostasis, and to elucidate the molecular mechanisms of microbiota and chronic stress on oral homeostasis. To this end, we compared the morphological structure of the tongue between GF and specific pathogen-free (SPF) mice and clarified the effects of microbiota and chronic stress on the biological function of oral tissues based on the integrated analysis of transcriptomics, proteomics and metabolomics.

## Material and methods

### Animal

Eight male Kunming mice in a specific pathogen-free environment and eight male GF mice in a germ-free environment were obtained from the NHC Key Laboratory of Diagnosis and Treatment on Brain Functional Diseases. All procedures involving animals were carried out in accordance with the guidelines of the Institutional Animal Care and Use Committee (IACUC) of Chongqing Medical University and ethical approval was obtained from the Laboratory Animal Ethics Committee of Chongqing Medical University (Approval Number: 2021(063)). Additionally, we have complied with the Animal Research: Reporting *In Vivo* Experiments (ARRIVE) 2.0 guidelines.

### Chronic stress

**Figure 1:**
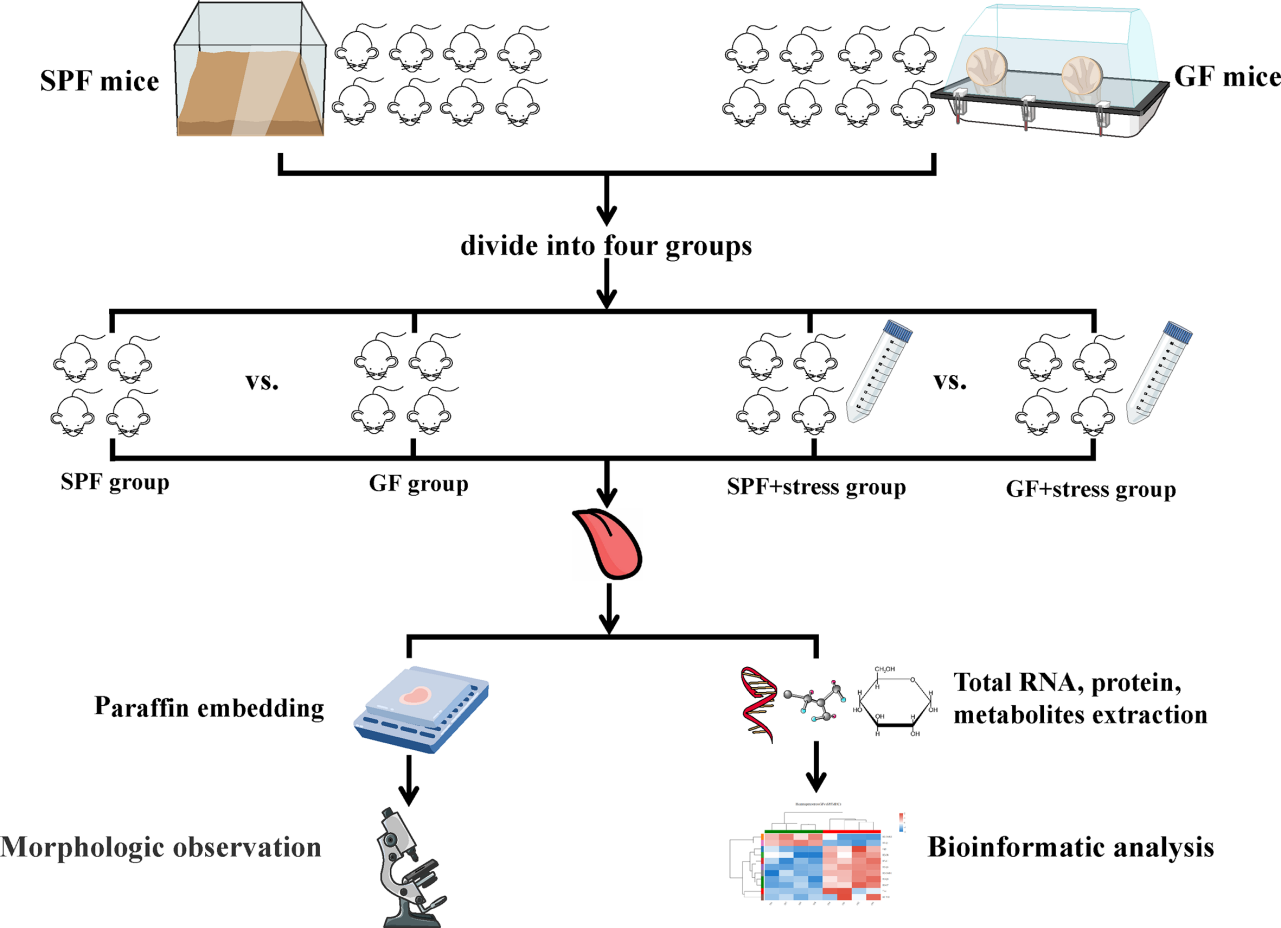
Flowchart of this study. (n=4/SPF group; n=4/GF group; n=4/SPF + chronic stress group; n=4/GF + chronic stress group). SPF mice, specific pathogen-free mice; GF mice, germ-free mice.

All mice were adaptively fed for one week before the formal experiment and a suitable number of mice were matched according to weight and age by statistical analysis. Half of the SPF mice and GF mice were confined to plastic tubes slightly larger than themselves for 2-4 hours a day, and the other mice were housed in a conventionally suitable environment (temperature of 23°C, humidity of 55%, day/night cycle of 12h/12h) every day. The experimental procedure is shown in **Figure 1**.

### Integrated analysis of transcriptomics, proteomics, and metabolomics

Metascape (https://metascape.org/gp/index.html#/main/) was used for the integrated analysis of differentially expressed genes (DEGs) and differentially expressed proteins (DEPs), and the biological enrichment pathways and functions of the above differentially expressed molecules were identified by Gene Ontology (GO), Kyoto Encyclopedia of Genes and Genomes (KEGG) and Reactome based on *p* < 0.05. Furthermore, MetaboAnalyst5.0 (http://www.metaboanalyst.ca/MetaboAnalyst/) was applied to the integrated analysis of transcriptomics, proteomics, and metabolomics. All identified differentially expressed molecules underwent to KEGG pathway enrichment analysis, and the top-ranked pathways were listed according to FDR values (FDR < 0.05). Molecular network analysis was then performed using Ingenuity Pathway Analysis (IPA, http://www.ingenuity.com). Statistical significance was set at an FDR < 0.05 in all analyses, and the top two in molecular networks were selected.

### Statistical analysis

All statistical operations were processed by the IBM SPSS Statistics (Version 26; IBM Corporation, Armonk, NY, USA) and GraphPad Prism 8 (GraphPad Software, San Diego, CA, USA). Quantitative data were expressed by mean ± standard deviation. The student’s t-test (two-tailed) was used to calculate the significance of the difference between the two groups of samples, and one-way ANOVA was conducted on multiple groups. A *p*-value < 0.05 was considered as statistically significant.

The detailed protocols of the following experiment are shown in the Supplementary materials:

H & E staining

Quantitative PCR

Transcriptomics Analysis

Proteomics Analysis

Metabolomics Analysis

## Results

1. Morphological observation indicates that microbiota and chronic stress affect the epithelial structure of the tongue

**Figure 2:**
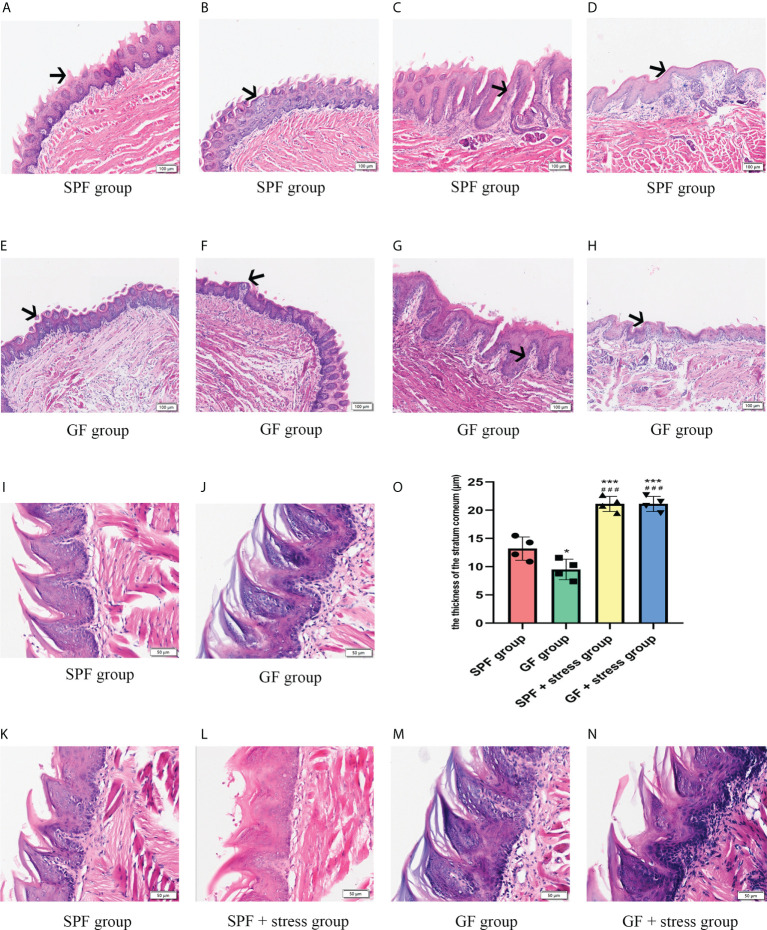
Microbiota regulates the morphological structure of the lingual epithelium. **(A, B)** HE staining of the anterior and middle regions of the lingual dorsum of SPF mice. The black arrows in Figures A and B represent the Fip and Fup on the lingual dorsum of SPF mice, respectively. **(C, D)** HE staining of the posterior region of the lingual dorsum in SPF mice. The black arrows in Figures C and D represent the Fop and Cip on the lingual dorsum of SPF mice, respectively. **(E, F)** HE staining of the anterior and middle regions of the lingual dorsum of GF mice. The black arrows in Figures E and F represent the Fip and Fup on the lingual dorsum of GF mice, respectively. **(G, H)** HE staining of the posterior region of the lingual dorsum in GF mice. The black arrows in Figures G and H represent the Fop and Cip on the lingual dorsum of GF mice, respectively. **(I)** The epithelial structure of Fip on the lingual dorsum of SPF mice. **(J)** The epithelial structure of Fip of the lingual dorsum of GF mice. **(K)** The morphological structure of tongue epithelium in SPF mice. **(L)** The morphological structure of tongue epithelium in SPF mice under chronic stress. **(M)**The morphological structure of tongue epithelium in GF mice. **(N)** The morphological structure of tongue epithelium in GF mice under chronic stress. **(O)** The bar graph of stratum corneum thickness of tongue epithelium in four groups of mice. Fip, filiform papillae; Fup, fungiform papillae; Fop, foliate papillae; Cip, circumvallate papillae. Scale bar: 100 or 50μm. The experiments were performed at least three times independently. *p < 0.05 vs SPF group; **p < 0.01 vs SPF group; ***p < 0.001 vs SPF group; #p <0.05 vs GF group; # #p < 0.01 vs GF group; # # #p < 0.001 vs GF group.

Morphological observation showed there were some similarities and differences in the structure of tongues between SPF and GF mice. The similarity was that the distribution of different types of lingual papilla in the dorsal lingual epithelium of SPF and GF mice was roughly the same (**Figure S1**). The difference was that the morphological structure of the tongue epithelium of was clearly visualized in SPF mice compared with GF mice, while the epithelial structure of GF mice was disordered, especially between stratum spinosum and basal layer (**Figures 2A–H**). Additionally, filiform papillae in GF mice were more slender and uneven than in SPF mice (**Figures 2I, J** **)**. There were no significant changes in the morphology of tongue in the two kinds of mice after chronic stress. Interestingly, we found that chronic stress led to the increase in the thickness of the epithelial stratum corneum of the tongue in both GF and SPF mice, especially in the filiform papillae (**Figures 2K–N**). The stratum corneum thickness of tongue epithelium was compared among the four groups, and it was statistically confirmed that chronic stress could thicken the stratum corneum of the tongue epithelium in both two kinds of mice (**Figure 2O**).

2. Multi-omics analysis reveals the influence of microbiota on oral homeostasis

**Figure 3:**
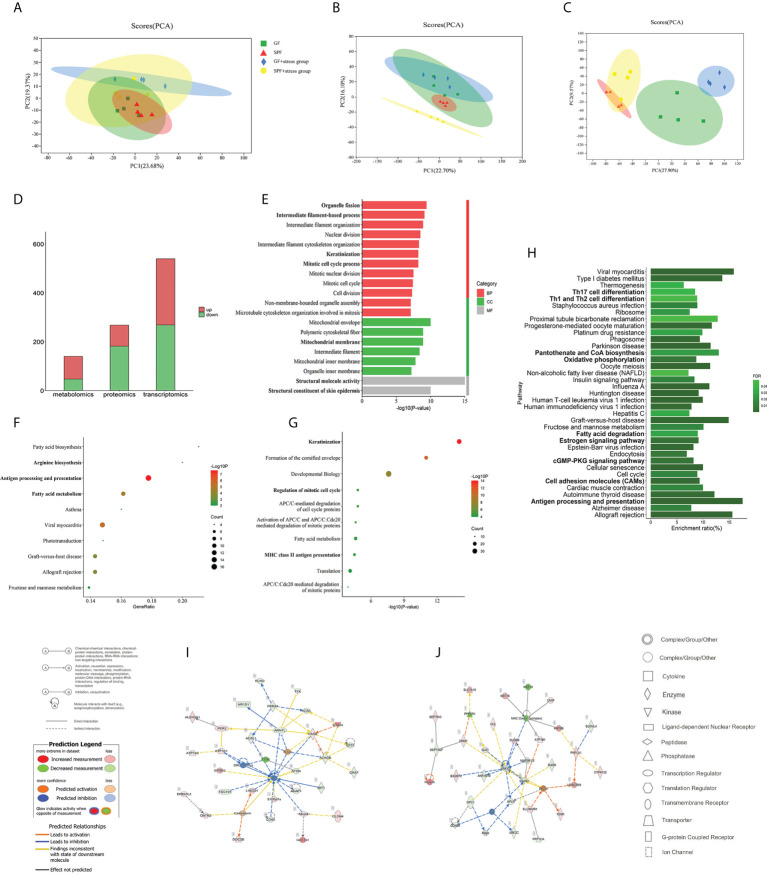
Multi-omics analysis reveals that microbiota can regulate oral homeostasis. **(A–C)** PCA of the transcriptomics, proteomics, and metabolomics of the tongue in SPF and GF mice. **(D)** Histogram showing the number of differentially expressed molecules in the tongues of SPF and GF mice during multi-omics analysis. **(E–G)** GO/KEGG/Reactome enrichment analysis of integrated transcriptomics and proteomics of the tongue in SPF and GF mice. **(H)** KEGG pathway enrichment analysis of multi-omics of the tongue in SPF and GF mice. **(I, J)** IPA of multi-omics of the tongue in SPF and GF mice. The bold fonts represent the molecules of particular interest in the enrichment pathway. PCA, Principal Component Analysis; IPA, Ingenuity Pathway Analysis; GO, Gene Ontology; KEGG, Kyoto Encyclopedia of Genes and Genomes.

A comparison of integrated multi-omics analysis of the tongue between SPF and GF mice reflected the molecular mechanism of microbial regulation on oral homeostasis. Principal Component Analysis (PCA) of transcriptomics, proteomics and metabolomics showed good biological duplication within the same group and clear segregation between the GF and SPF group, especially in the metabolomics (**Figures 3A–C**), which ensured the reliability of the subsequent analysis. Additionally, we performed inter-group Venn analysis and showed the co-expressed and specially expressed transcripts, proteins, and metabolites between GF and SPF group. (**Figure S2**).

We analyzed DEGs in the tongue tissues of SPF and GF mice using DESeq2, and a total of 540 DEGs were identified (using the criteria false discovery rate, [FDR] < 0.05 and fold change, [|FC|] > 2). We found that 271 and 269 DEGs were up-regulated and down-regulated, respectively (**Figure S3A**). For DEPs, a total of 268 proteins were obtained based on thresholds of fold change >1.2 or 0.83, and *p* < 0.05, including 86 upregulated and 182 downregulated proteins (**Figure S3B**). 140 differential expressed metabolites (*p* < 0.05 and VIP > 1) were detected in the tongue tissues between GF and SPF mice, including 93 upregulated and 47 downregulated metabolites (**Figure S3C**). The numbers of differentially expressed molecules in the multi-omics analysis are shown in **Figure 3D**.

3. An integrated bioinformatics analysis of the tongue tissues between SPF and GF mice suggests a mechanism underlying the regulatory role of microbiota on oral homeostasis

To conduct a bioinformatics analysis of the above differentially expressed molecules, the biological enrichment pathways and functions of the above DEGs and DEPs were first identified by Gene Ontology (GO), Kyoto Encyclopedia of Genes and Genomes (KEGG) and Reactome (**Table S1**). Among the 670 GO items, the top 20 terms were “Structural molecule activity”, “Structural constituent of skin epidermis”, “Mitochondrial envelope”, “Organelle fission”, and so on (**Figure 3E**). Interestingly, the above items were mainly involved in mitochondrial metabolism, cell division, and keratinization. There were 66 and 117 items in KEGG and Reactome pathway enrichment analysis, and the top 10 items are shown in **Figures 3F, G**, including significant enrichment in “Antigen processing and presentation”, “Fatty acid (FA) metabolism”, and “Keratinization”.

To comprehensively explore the effect of microbiota on oral homeostasis, we performed an integrated analysis of the differentially expressed molecules, which yielded 289 enriched pathways (**Table S2**). After screening, a total of 37 pathways were statistically significant, and the results showed significant enrichment in functions, including “Th17 cell differentiation”, “Th1 and Th2 cell differentiation”, “Oxidative phosphorylation (OXPHOS)”, “Cell adhesion molecules (CAMs)”, and “Antigen processing presentation” (**Figure 3H**). We then identified the molecular networks using Ingenuity Pathway Analysis and selected the top two. The results indicated that the microbiota might play an important regulatory role in oral homeostasis by activating immune response and inflammatory reaction (**Figures 3I, J**; **Table S3**).

4. Multi-omics analysis reveals the influence of chronic stress on oral homeostasis

**Figure 4:**
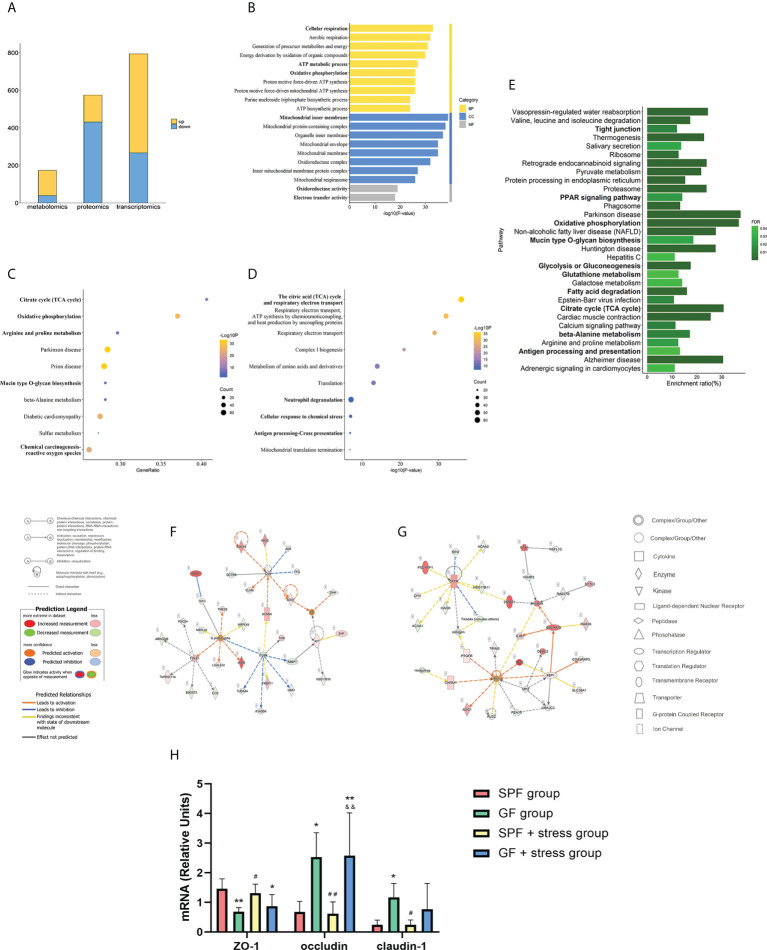
Multi-omics analysis suggests that chronic stress may enhance the regulation of microbiota on oral homeostasis. **(A)** Histogram showing the number of differentially expressed molecules during the tongues of SPF and GF mice in multi-omics analysis under chronic stress. **(B–D)** GO/KEGG/Reactome enrichment analysis of combined transcriptomics and proteomics of the tongue in SPF and GF mice under chronic stress. **(E)** KEGG pathway enrichment analysis of multi-omics of the tongue in SPF and GF mice under chronic stress. **(F, G)** IPA of multi-omics of the tongue in SPF and GF mice under chronic stress. **(H)** The bar graph of mRNA expression representing oral epithelial permeability in four groups of mice. The bold fonts represent the molecules of particular interest in the enrichment pathway. Scale bar: 50μm. The experiment of qPCR was performed at least three times independently. *p < 0.05 vs SPF group; **p < 0.01 vs SPF group; ***p < 0.001 vs SPF group; #p <0.05 vs GF group; ##p < 0.01 vs GF group; ###p < 0.001 vs GF group; &p < 0.05 vs SPF + stress group; &&p < 0.01 vs SPF + stress group; &&&p < 0.001 vs SPF + stress group.

To investigate the effects of chronic stress on oral homeostasis from a multi-omics perspective, the PCA results showed clear segregation between two types of mice under chronic stress, and the trend was more obvious than in the non-chronic stress state (**Figures 3A–C**). The above results indicated that there was clear segregation between the GF and SPF group under chronic stress, especially in proteomics and metabolomics results. The inter-group Venn analysis was performed to show the co-expressed and specially expressed transcripts, proteins, and metabolites between GF and SPF group under the chronic stress (**Figure S4**). The intersection of genes between SPF and GF mice under chronic stress yielded 794 DEGs (FDR < 0.05 and |FC| > 2) (**Figure S5A**), among which 527 DEGs were up-regulated and 267 DEGs were down-regulated (**Figure 4A**). The number of differentially expressed proteins and metabolites of tongues between GF and SPF mice under chronic stress was 574 and 173, respectively (*p* < 0.05) (**Figures S5B, C**, **4A**).

5. The integrated bioinformatics analysis of the tongue between SPF and GF mice under chronic stress suggests the molecular mechanism of chronic stress on oral homeostasis

We performed an integrated transcriptomics and proteomics analysis of the tongue between SPF and GF mice under chronic stress (**Table S4**), and GO, KEGG, and Reactome enrichment analyses were used to identify enriched pathways and functions of DEGs and DEPs under chronic stress. GO enrichment analysis yielded 932 items, among which “Cellular respiration”, “Mitochondrial inner membrane”, and “Oxidoreductase activity” were the top GO terms for biological process, cellular component, and molecular function, respectively (**Figure 4B**). KEGG and Reactome pathway enrichment analysis yielded 68 and 197 items, respectively, and the top 10 items are shown in **Figures 4C, D**. KEGG enrichment analysis showed significant enrichment in “Citrate cycle (TCA)”, “Oxidative phosphorylation”, and “Mucin type O-glycan biosynthesis”, while Reactome pathway enrichment analysis indicated significant differences in “Antigen processing - Cross presentation”, “Cellular response to chemical stress”, and “Neutrophil degranulation” between the two groups.

To comprehensively explore the mechanisms of chronic stress on oral homeostasis, we integrated the data from transcriptomics, proteomics and metabolomics analysis, and the results indicated that chronic stress mainly affected biological functions, including mitochondrial metabolism, epithelial adhesion and immune response in oral homeostasis (**Figure 4E**; **Table S5**). “OXPHOS”, “Glycolysis or gluconeogenesis”, “Glutathione metabolism”, “FA degradation”, “Citrate cycle”, and “beta-alanine metabolism” reflected the mitochondrial metabolic process, while epithelial adhesion was associated with “Tight junction”, “PPAR signaling pathway”, and “Mucin type O-glycan biosynthesis”. Additionally, Ingenuity Pathway Analysis indicated that chronic stress might affect oral homeostasis by activating inflammatory cascade reaction and immune response (**Figures 4F, G**; **Table S6**). In order to better demonstrate the effects of microbiota and chronic stress on oral homeostasis, the mRNA expression of epithelial permeability indicators was detected in tongue tissues of the four groups of mice. The results showed that compared with SPF mice, the expression of ZO-1 in the tongue tissue of GF mice decreased, while the expression of occludin and claudin-1 increased, and chronic stress caused similar changes in oral epithelial permeability (**Figure 4H**). Overall, our results suggest that chronic stress can affect the structural remodeling and biological function of the oral epithelium, thus enhancing the regulatory role of microbiota on oral homeostasis.

## Discussion

To the best of our knowledge, this is the first study to comprehensively analyze the molecular regulatory mechanisms of microbiota and chronic stress on oral homeostasis by comparing the morphological structure and integrating multi-omics data of the tongue between SPF and GF mice. We found that the microbiota could affect structural remodeling of the oral epithelium, which was mainly reflected in keratinization and cellular adhesion, and the microbiota may regulate oral homeostasis by mitochondrial metabolic process and immune response. Additionally, chronic stress can enhance these regulatory processes.

Herein, we found that the microbiota can regulate oral homeostasis by affecting structural remodeling. As the structural basis of the oral mucosa, the integrity of the epithelial barrier is a firewall against pathogenic microbiota. Indeed, oral epithelial morphology also embodies the integrity of the epithelial barrier. In this study, we found that the morphological structure of oral epithelium in GF mice was more disordered in SPF mice, and the lingual papilla was more slender and uneven, similar to nasal and intestinal mucosal epithelium structure (2, 3). Proper structure and function of the oral epithelium are essential for maintaining symbiosis of bacteria and protecting the host from infection, especially for keratinization. Indeed, epithelial cells participate in the effective defense against microbiota invasion. A study found that the keratinization of the oral mucosa of baboons was closely related to the number of bacteria (13). In addition to keratinization, the expression of CAMs also plays an important role in epithelial permeability. An epithelium with poor permeability confers an appropriate protective effect against pathogen infections, and the high expression of CAMs reflects the repeated resistance of the mucosal epithelium to the attack of pathogen infections (14). The expression of mucosal Adressin cell adhesion molecule 1 (MAdCAM-1) at birth was more prevalent in the intestinal mucosal blood vessels of CV mice than in GF mice (15). In oral mucosa, it was also found that the microbiota limited the absorption of foreign pathogens in the mucosal barrier by increasing the expression of epithelial-adhesive proteins to support the protective mucosal layer (16), which was consistent with the mRNA expression of epithelial adhesion molecule in our study. The enhanced permeability of the tongue epithelium in GF mice suggested that microbiota could activate the protective mechanism of the oral epithelial barrier. Moreover, human keratinocytes exposed to *Porphyromonas gingivalis* (Pg) showed low expression of connexin and Grainyhead-like 2 (*Grhl2*), and *Grhl2* conditional knockout (KO) mice showed increased epithelial infiltration of oral bacteria compared with wild-type mice, which could disrupt the epithelial barrier, thereby leading to the aggravation of periodontal disease (17). Our results suggested that hyper-keratinization of epithelial cells and adhesion change of endothelial cells were important manifestations of host anti-microbial defense.

Our integrated omics analysis revealed that microbiota could regulate the immune response in oral homeostasis. Oral epithelium can trigger inflammatory cascade signals and immune responses during microbial imbalance. Firstly, EGF/EGFR, as a known upstream activator of both the pro-survival phosphoinositide 3-kinase/Akt and proinflammatory mitogen-activated protein (MAP) kinase pathways, could protect oral epithelia from barrier damage (18–20). Mechanically, EGF/EGFR can play a positive regulatory role on the permeability of mucosal barrier by regulating the integrity of tight junction proteins (21). It is worth noting that EGF can change the ability of microbiota to colonize the intestinal and respiratory mucosa and prevent barrier defects induced by pathogenic microbiota (22, 23). Additionally, it has found that the severity of oral mucositis was positively correlated with the content of EGF in saliva (24). In this study, our integrated analysis indicated that EGF/EGFR could be activated by microbiota and chronic stress to regulate oral homeostasis. It is well-established that *Streptococcus* and *Veillonella* in oral flora can activate a series of inflammatory tandem pathways represented by MAPK and PI3K-Akt-mTOR (25, 26). The microbiota can induce host immune defense by direct contact with microbiota or indirectly activating inflammatory cytokines, and this process is environmentally adaptive. It has been reported that T-cells response to *Akkermansia muciniphila* in a sterile environment could be limited to the activation of T follicular helper cells. In contrast, in an inflammatory environment, various manifestations such as Th17 cell differentiation have been observed (27). It has been shown that *Bacteroides Fragilis* could enhance the differentiation of CD4+ T cells into T helper cells 1 (Th1) and Th2 (28), and the above phenomenon of helper T cell skewing was similar to our results. In the present study, we revealed that the microbiota affects oral homeostasis *via* an immune response by activating inflammatory cascade signals, which are adaptive changes after the microbiota competes with the host.

**Figure 5:**
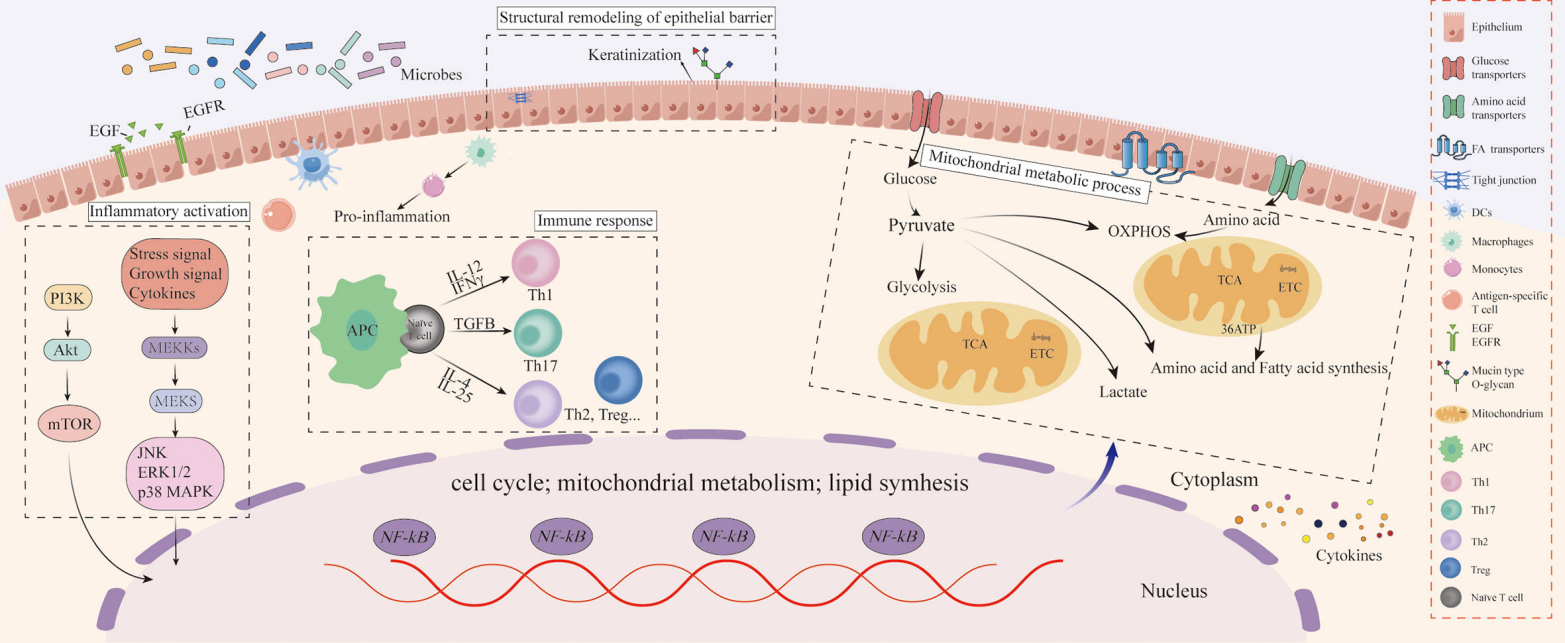
Schematic diagram of oral epithelial barrier resistance to microbial invasion.

Furthermore, our results demonstrated that mitochondrial metabolism might also be a major regulator of microbial-mediated oral homeostasis. Metabolic adaptation is the basis of cell life activities, and the mitochondrial metabolic process can regulate microbial-mediated oral homeostasis by affecting immune cell differentiation (29). The metabolic processes of OXPHOS, TCA cycle, and nucleotide biosynthesis are strongly activated in activated naïve and memory T cells, while aerobic glycolysis is a marker of activated CD8+ T cells (30). In addition, it has been established that Fatty acid (FA) provides energy for cell metabolism through FA oxidation and closely regulates the homeostasis of immune cells (31). The results of our multi-omics analysis suggested that microbiota may regulate oral homeostasis by changes in the mitochondrial metabolic process, thus affecting the epithelium’s inflammatory activation and immune response of. Experiments with *Staphylococcus aureus* infection of human keratinocytes demonstrated a positive correlation between glycolysis and innate immunity of epithelial cells (32). Thus, upregulation of glycolysis is more likely to occur in an inflammatory environment (33), whereas OXPHOS positively correlates with anti-inflammatory activation (34). Additionally, membrane-associated protein can reportedly promote the uptake of FA to meet the host immune cells’ nutritional supply and energy requirements (35), consistent with our results. Collectively, specific metabolic changes may adapt to different functional outputs, and the mitochondrial metabolic process could be an adaptive adjustment of host defense pathogens in the epithelium (**Figure 5**).

Herein, we explored the role of chronic stress in oral homeostasis. One the one hand, chronic stress could regulate the morphological structure of the oral epithelium. On the other hand, we found significant differences in the expression of tight junction and mucin-type O-glycan biosynthesis in the tongues of GF and SPF mice, consistent with the literature (36). In this respect, the loss of O-glycosyltransferase in oral mucus-secreting cells led to changes in oral microbial composition, suggesting that O-glycosylation regulated microbial composition and the stability of colonization (36). Mucin is a protein with high-density O-glycosylated serine and threonine residue domains, closely related to microbial dysregulation in the mucosa (37). In saliva, N- and O-terminal glycan structures may act as bait-binding receptors to competitively inhibit pathogen adherence to oral mucosal surfaces (38). A study found that mucin O-glycan was involved in the microbial-mediated immune tolerance in intestinal mucosa during the development of microbial-induced gastrointestinal disease. Interestingly, glycosylation patterns in the distal intestinal epithelium of mice have changed under psychological stress, and this region became a marker for resistance to microbial attack (39). In addition to mucin-type O-glycan, tight junctions often act as important regulatory components of transmembrane pattern recognition receptors such as Toll-like receptors to protect the epithelium from exogenous pathogens (40). Compared with SPF mice, the expression of junctional complex molecules in colon epithelial cells of GF mice was decreased, which was consistent with the expression of epithelial permeability factors in tongue tissues of different groups in our study. Oral administration of indole-containing capsules increased the expression of tight junction-related molecules in the colon epithelium of GF mice, resulting in higher resistance to dextran sodium sulfate (DSS)-induced colitis in GF mice (41). Overall, chronic stress can regulate the biological function by modifying the epithelial barrier structure, thus maintaining oral homeostasis against adverse microbial invasion.

There were still some limitations in this study. First, the sample size included in this study was small, which increased the experimental error to some extent. Besides, gender and species heterogeneity in microbial-mediated oral homeostasis emphasize the need for further investigation to understand the underlying mechanisms of pathogenic microbiota and epithelial barrier interactions. Additionally, other oral tissues can be applied for more research. Finally, in addition to chronic stress, more stress models should be considered in further studies.

In conclusion, we investigated the morphological structure and integrated the transcriptomics, proteomics, and metabolomics data to study the microbiota’s effects and mechanisms on oral homeostasis. The findings of this study suggest that microbiota may regulate oral homeostasis by affecting structural remodeling and biological function. Furthermore, chronic stress can enhance the regulatory role of microbiota on oral homeostasis by affecting the mitochondrial metabolic process and immune response. This study contributes to existing knowledge of microbiota, and reveals the limitations of previous studies investigating the biological effects of microbiota on oral homeostasis.

## Data availability statement

The original contributions presented in the study are included in the article/**Supplementary Material**. Further inquiries can be directed to the corresponding authors.

## Ethics statement

The animal study was reviewed and approved by the Institutional Animal Care and Use Committee (IACUC) of Chongqing Medical University and ethical approval was obtained from the Laboratory Animal Ethics Committee of Chongqing Medical University (Approval Number: 2021(063)).

## Author contributions

HL contributed to design, and interpretation, drafted and critically revised the manuscript; LiY and XZ contributed to data acquisition and analysis; JP, YiL, and HW contributed to conception and critically revised the manuscript; LuY , FL, and SL contributed to data analysis and interpretation; YZ, YaL, and PX contributed to design and interpretation; JP and XJ contributed to design and critically revised the manuscript. All authors gave final approval and agree to be accountable for all aspects of the work.

## Funding

The study was supported by grants from the National Natural Science Foundations of China (No. 81870775, 81500855, and 81771081).

## Conflict of interest

The authors declare that the research was conducted in the absence of any commercial or financial relationships that could be construed as a potential conflict of interest.

## Publisher’s note

All claims expressed in this article are solely those of the authors and do not necessarily represent those of their affiliated organizations, or those of the publisher, the editors and the reviewers. Any product that may be evaluated in this article, or claim that may be made by its manufacturer, is not guaranteed or endorsed by the publisher.
