# Supplementary material for: Multi-omics analysis reveals the effects of microbiota on oral homeostasis
Source: Front Immunol. 2022 Sep 20;13:1005992. doi: 10.3389/fimmu.2022.1005992 (PMC9533175; doi:10.3389/fimmu.2022.1005992)

Materials and Methods

H & E staining

Fresh tongue tissues of GF and SPF mice were fixed with 4% formalin, dehydrated, embedded in paraffin, and cut into 5 µm thick sections. HE staining was performed as follows: hematoxylin was used to stain the nucleus for 5 minutes. The sections were differentiated with hydrochloric acid ethanol and stained with eosin. After dehydration, the sections were sealed with neutral balsam. All images were scanned by Olympus Image Viewer software.

Quantitative PCR

In short, total RNA of tongue tissues was extracted using the RNA extraction kit (Beyotime Biotechnology, China). After the content of RNA was determined, it was reversely transcribed into cDNA (MCE, USA), followed by PCR amplification to obtain the quantitative value of gene content (TaKaRa, Japan). PCR primer sequences contained: ZO-1, 5’-GCTGTGGGTAACTCCATCCT-3’ (sense) and 5’-GGCTGACAGTGGAAGTAGCA-3’ (antisense); occluding, 5’-GGAGTTTCAGGTGAATGGGTCA-3’ (sense) and 5’-AAATGTCCAGGCTCCCAAGA-3’ (antisense); claudin-1, 5’-CCCTTCAGCAGAGCAAGGTT-3’ (sense) and 5’-TAGGGCAACCAAGTGCCTTT-3’ (antisense); β-actin, 5’-AGAAAATCTGGCACCACACCT-3’ (sense) and 5’-GATAGCACAGCCTGGATAGCA-3’ (antisense). The expression of all above genes was normalized to β-actin and determined by the method of 2-ΔΔCT.

Transcriptomics Analysis

RNA extraction and RNA Sequencing

Total RNA was extracted from the tissue using TRIzol® Reagent (Thermo Fisher Scientific, Shanghai, China) according to the manufacturer’s instructions (Invitrogen, CA) and genomic DNA was removed using DNase I (TaKara, Japan). The RIN value was measured by Agilent 2100 (Agilent Technologies Inc., California, USA), and only high-quality RNA samples (OD260/280 between 1.8~2.2, the total volume of RNA > 1ug, and the concentration of RNA > 50ng/μL) were used to construct sequencing library. The RNA-seq transcriptome library was prepared following using the TruSeqTM RNA sample preparation Kit from Illumina (San Diego, CA, USA). First, messenger RNA was isolated according to the polyA selection method by oligo (dT) beads and then fragmented by fragmentation buffer. Then, a cDNA strand was synthesized by reverse transcriptase using mRNA as the template (TaKara, Japan), and double-stranded cDNA was synthesized using a SuperScript double-stranded cDNA synthesis kit (Invitrogen, CA) with random hexamer primers (Illumina). Then the synthesized cDNA was subjected to end-repair, phosphorylation and ‘A’ base addition according to Illumina’s library construction protocol. Libraries were size selected for cDNA target fragments of 300 bp on 2% Low Range Ultra Agarose followed by PCR amplified using Phusion DNA polymerase (NEB) for 15 PCR cycles. After quantification by TBS380, paired-end RNA-seq sequencing library was sequenced with the Illumina HiSeq xten/NovaSeq 6000 sequencer (2 × 150bp read length). The reference genome source was <http://asia.ensembl.org/Mus_musculus/Info/Index>.

Read mapping, and differentially expressed gene analysis

The raw paired-end reads were trimmed and quality controlled by SeqPrep (<https://github.com/jstjohn/SeqPrep>) and Sickle (<https://github.com/najoshi/sickle>) with default parameters. Then clean reads were separately aligned to the reference genome with orientation mode using HiSat2 (<http://ccb.jhu.edu/software/hisat2/index.shtml>) software. The mapped reads of each sample were assembled by StringTie (<https://ccb.jhu.edu/software/stringtie/index.shtml?t=example>) in a reference-based approach. To identify differentially expressed genes (DEGs) between two different samples, the expression level of each transcript was calculated according to the transcripts per million reads (TPM) method. RSEM (<http://deweylab.biostat.wisc.edu/rsem/>) was used to quantify gene abundances. Differential expression analysis was performed using the DESeq2 with Q value ≤ 0.05, DEGs with |log2FC| > 1 and Q value ≤ 0.05 were considered as significant differentially expressed genes.

Proteomics Analysis

Total protein extraction, protein digestion, peptide desalination and quantification

An appropriate amount of protein lysis solution (1% SDS, 8 M urea, cocktail) was added to the frozen tongue tissue, and the ratio of sample to protein lysis was 1:10. The solution was centrifuged at 12000g for 20 min at 4 ℃, and supernatant was collected. Finally, protein concentrations were determined by Bicinchoninic acid (BCA) method by the BCA Protein Assay Kit (Beyotime Biotechnology, Shanghai, China) and protein extraction quality was performed by SDS-PAGE (Beyotime Biotechnology, Shanghai, China). Protein digestion was performed according to the standard procedure. Briefly, for each sample tube containing 100 μg protein, appropriate Tris(2-carboxyethyl) phosphine (TCEP) was added to obtain a final concentration of 10 mM, and incubated at 37 °C for 60 min. An appropriate amount of Iodoacetamide (IAM) was added to obtain a final concentration of 40 mM and allowed to react for 40 min in the dark. Six volumes of cold acetone were added to the sample tube. After inverting the tube three times, it was incubated the tube at –20 °C until a precipitate was formed (~4 h). The pre-cooling acetone was removed by centrifugation at 10000 g for 20 min, and the precipitated protein was resuspended with100 µl 100 mM TEAB Buffer. The trypsin solution was added to each sample tube according to the ratio of 1:50, and the tubes were incubated at 37 °C overnight. The peptide was desalted with Sep-Pak (Thermo Fisher Scientific, USA) and quantified by a peptide quantification kit (Thermo, Cat.23275). Loading buffer was added to each tube to prepare samples for mass spectrometry analysis, and the concentration of each sample was 0.5 µg/µl.

Mass spectrometry analysis and sequence database searching

Experiments were performed on a Q Exactive mass spectrometer coupled with Easy-nLC 1200 (Thermo, USA). Each peptide sample was injected for nanoLC-MS/MS analysis. The sample was loaded onto a C18 reversed-phase column (75 μm x 25 cm, Thermo, USA) in buffer A (2% acetonitrile and 0.1% Formic acid) and separated with a linear gradient of buffer B (80% acetonitrile and 0.1% Formic acid) at a flow rate of 300 nl/min. The electrospray voltage of 1.8 kV versus the inlet of the mass spectrometer was used. Q Exactive mass spectrometer was operated in the data-dependent mode to switch automatically between MS and MS/MS acquisition. Survey full-scan MS spectra (m/z 350-1300) were acquired with a mass resolution of 70K, followed by twenty sequential high energy collisional dissociation (HCD) MS/MS scans with a resolution of 17.5K. In all cases, one microscan was recorded using dynamic exclusion of 18 seconds. MS/MS spectra were searched using Proteome Discoverer ^TM^ Software 2.2 software against the Mus musculus Proteomic database (https://www.uniprot.org/taxonomy/10090). The parameter settings were as follows: tryptic digestion with up to two missed cleavages, carbamidomethylation of cysteines as fixed modification, and oxidation of methionines and protein N-terminal acetylation as variable modifications. Peptide spectral matches were validated based on q-values at a 1% false discovery rate (FDR).

The student's T test was used to calculate the significant of the difference between samples based on the protein abundance information obtained from the Mus musculus Proteomic database. The thresholds of fold change > 1.2 or < 0.83, and *p*-value < 0.05 were used to identify differentially expressed proteins (DEPs).

Metabolomics Analysis

Metabolite extraction and LC–MS metabolite analysis

50mg of tongue tissue were accurately weighed, and the metabolites were extracted using a 400 µL methanol:water (4:1, v/v) solution. The mixture was treated by High throughput tissue crusher Wonbio-96c (Shanghai wanbo biotechnology co., LTD) at 50 Hz for 6 min, followed by vortexing for 30s and ultrasound at 40 kHz for 30 min at 5℃. The samples were placed at -20℃ for 30min to precipitate proteins. After centrifugation at 13000g at 4℃ for 15min, the supernatant was carefully transferred to sample vials for LC-MS analysis. Chromatographic separation of the metabolites was performed on an ExionLCTMAD system (AB Sciex, USA) equipped with an ACQUITY UPLC BEH C18 column (100 mm × 2.1 mm i.d., 1.7 µm; Waters, Milford,USA). The UPLC system was coupled to a quadrupole-time-of-flight mass spectrometer (Triple TOFTM5600+, AB Sciex, USA) equipped with an electrospray ionization (ESI) source operating in positive mode and negative mode. Data acquisition was performed with the Data Dependent Acquisition (DDA) mode. The detection was carried out over a mass range of 50-1000 m/z.

Data processing and differential metabolites analysis

After UPLC-TOF/MS analyses, the raw data were imported into the Progenesis QI 2.3 (Nonlinear Dynamics, Waters, USA) for peak detection and alignment. Mass spectra of these metabolic features were identified by using the accurate mass, MS/MS fragments spectra and isotope ratio difference by searching in reliable biochemical databases such as the Human metabolome database (HMDB) (<http://www.hmdb.ca/>) and Metlin database (<https://metlin.scripps.edu/>). A multivariate statistical analysis was performed using ropls (Version1.6.2, http://bioconductor.org/packages/release/bioc/html/ropls.html) from Bioconductor on Majorbio Cloud Platform (<https://cloud.majorbio.com>). Variable importance in projection (VIP) was calculated in the OPLS-DA model. *P* values were estimated with paired Student’s t-test during one-dimensional statistical analysis. The metabolites with VIP > 1 and *p* < 0.05 were identified as significant.

Supplementary Figures

Supplementary Figure 1. (A-B) The landscape of the overall lingual papillae distribution in SPF and GF mice, respectively.


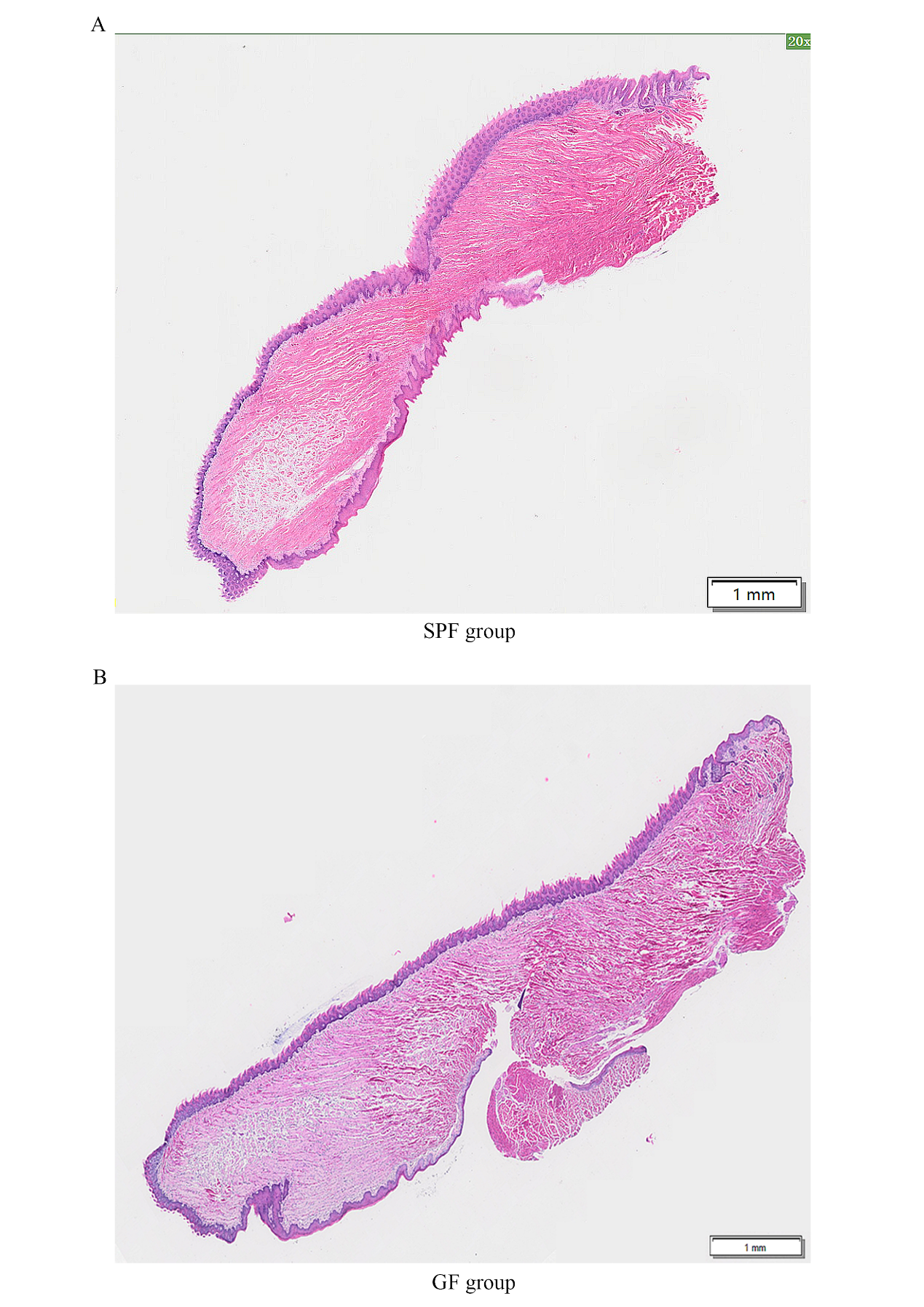


Supplementary Figure 2. (A-C) The inter-group Venn plot of transcriptomics, proteomics, and metabolomics analysis of the tongue in SPF and GF mice.


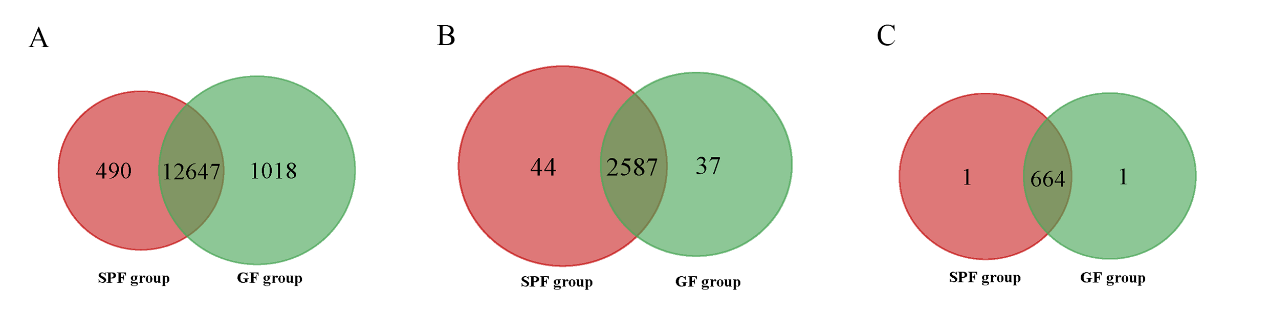


Supplementary Figure 3. (A-C) Volcano plot of the transcriptomics, proteomics, and metabolomics of the tongue in SPF and GF mice, respectively. Nodes represent differentially expressed molecules, the x-axis shows the log2FC, and the y-axis shows the −log10 (*p*-value). Red and green nodes represent upregulated and downregulated differentially expressed molecules, respectively.


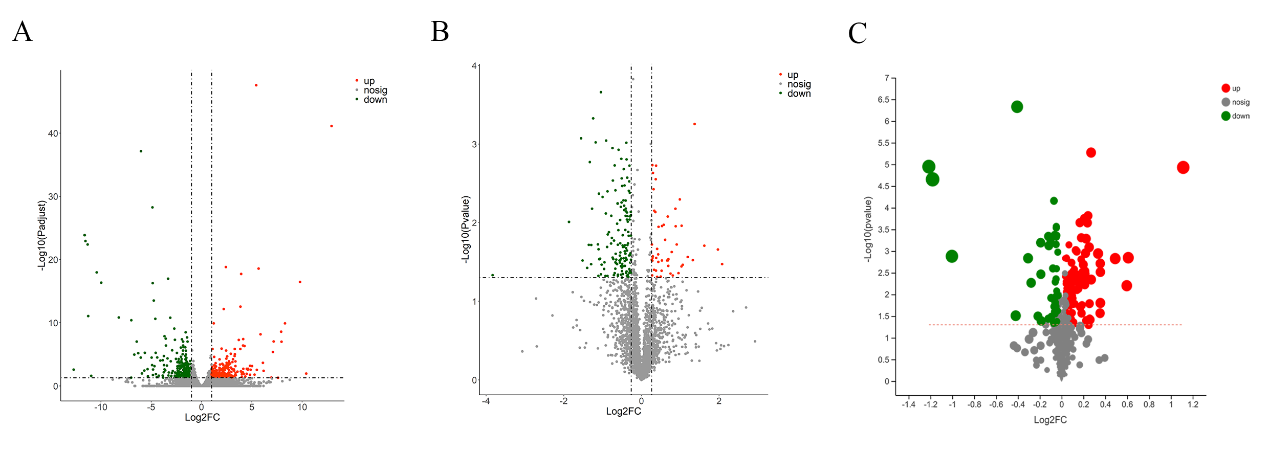


Supplementary Figure 4. (A-C) The inter-group Venn plot of transcriptomics, proteomics, and metabolomics analysis of the tongue in SPF and GF mice under chronic stress.


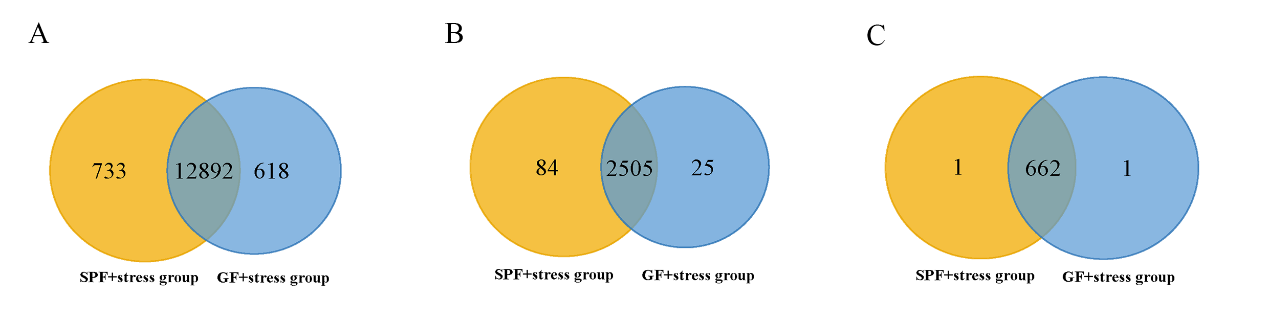


Supplementary Figure 5. (A-C) Volcano plot of the transcriptomics, proteomics, and metabolomics of the tongue in SPF and GF mice under chronic stress, respectively. Nodes represent differentially expressed molecules, the x-axis shows the log2FC, and the y-axis shows the −log10 (*p*-value). Red and green nodes represent upregulated and downregulated differentially expressed molecules, respectively.


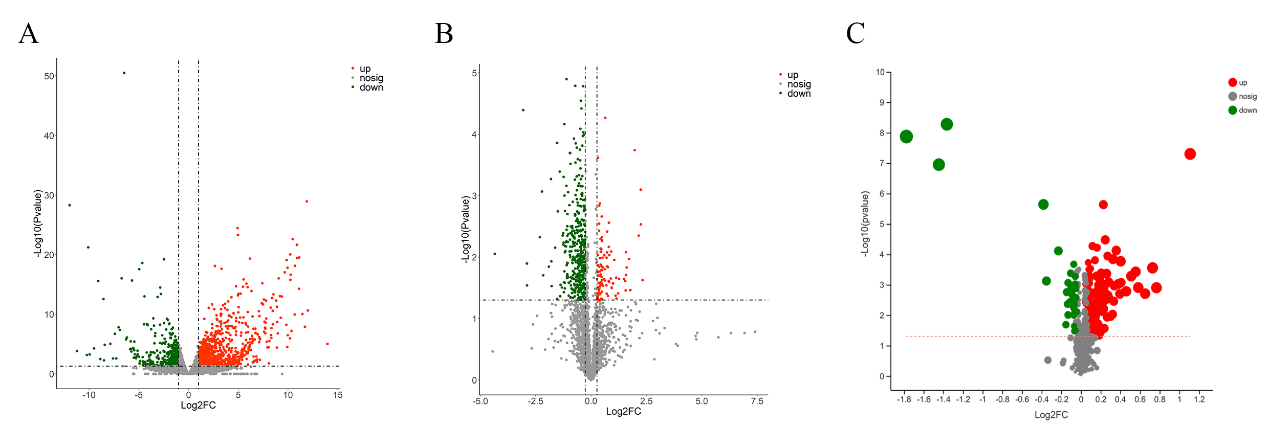

Supplement: Supplementary file 1 [file DataSheet_1.docx]
